# From Therapeutic Refusal to Gynecological Emergency: A Case Report on a Long-Standing Uterine Prolapse

**DOI:** 10.7759/cureus.97577

**Published:** 2025-11-23

**Authors:** Jorge Antunes, Mariana Monteiro, Cristina Bacelar

**Affiliations:** 1 Family Medicine, Unidade de Saúde Familiar (USF) Covelo, Unidade Local de Saúde (ULS) São João, Porto, PRT

**Keywords:** patient compliance, pelvic organ prolapse, pessary, primary health care, therapeutic refusal, uterine prolapse

## Abstract

Uterine prolapse is a type of pelvic organ prolapse (POP) that corresponds to the herniation with inferior displacement of the middle/apical compartment, in which the uterus descends from its normal position into or beyond the vaginal canal due to the weakening of the pelvic floor support structures. Its presentation can range from mild and asymptomatic forms to voluminous prolapse with functional impact and associated complications. This case illustrates a situation sparsely documented in the literature: the prolonged evolution of a uterine prolapse in a woman with multiple comorbidities, monitored in primary healthcare, who had previously refused corrective surgery in 2019. A 70-year-old woman, with a clinical history of one gestation and one eutocic delivery, menopause at 54 years (no hormone replacement therapy (HRT)), arterial hypertension, ischemic stroke (no identified sequelae), dyslipidemia, and obesity, was diagnosed with uterine prolapse since 2015. In July 2025, during a routine consultation and after direct questioning, she reported worsening symptoms due to increased exteriorization of the prolapse and episodes of scant abnormal uterine bleeding. Gynecological examination revealed a complete uterine prolapse (stage IV), non-reducible, with cervical erosion and active bleeding. Reduction was attempted without success. Local compression was applied, and she was referred to the gynecology emergency department, where, upon re-evaluation, she no longer presented active bleeding. Manual reduction of the uterine prolapse was performed, which recurred immediately upon standing. She was discharged with a referral back to her family physician, with instructions for referral to the outpatient gynecology clinic. At the outpatient gynecology clinic, she again refused surgery, opting for the placement of an 85 mm pessary. Thus, this case highlights the importance of longitudinal follow-up, objective examination, and active listening in general and family medicine, especially in patients with multiple comorbidities and previously postponed therapeutic decisions. Timely action in the face of warning signs and coordination with hospital care are essential to ensure safety and continuity of care.

## Introduction

The global prevalence of pelvic organ prolapse (POP) is high, and it is estimated that, on physical examination, the prevalence may reach 41.8% in women [1]. POP refers to the descent of one or more of the following compartments: anterior, middle/apical, or posterior. According to the literature, vaginal delivery and its effects on the pelvic floor represent the predominant etiological mechanism for the development of this condition. Additionally, factors such as advanced age, multiparity, obesity, a family history of prolapse, chronic constipation, and activities that repeatedly increase intra-abdominal pressure contribute to the risk of POP [2-4].

Estimating the true prevalence of uterine prolapse is challenging due to the influence of sociocultural, occupational, and racial factors, as well as variations in health-seeking behavior. Additionally, distinguishing specific rates of uterine prolapse is difficult, as most studies aggregate it with general POP. Nevertheless, current estimates suggest that prevalence based on reported symptoms is significantly lower (3-6%) than that detected upon clinical examination (41-50%) [5,6].

The pathophysiology of uterine prolapse reflects a reduction in the structural integrity of the pelvic floor, including the weakening of the levator ani muscles and pelvic fascia, thereby allowing the uterus to descend. Additional contributing factors include genetic predisposition, lifestyle, hormonal influences, and comorbidities affecting connective tissue strength [3,7-9].

The most common clinical presentation of uterine prolapse involves the sensation of a vaginal bulge accompanied by pelvic pressure. Associated symptoms often include increased urinary urgency or frequency, incomplete bladder emptying, and dyspareunia. These symptoms typically have a gradual onset and may exacerbate as the prolapse progresses. Evidence shows that symptom burden correlates with the severity of the prolapse [6]. Consequently, clinical presentation ranges from mild, asymptomatic cases to voluminous prolapse with significant functional impact and complications, such as hemorrhage, ulceration, or infection [5,6].

Furthermore, weakness of the pelvic floor attachments, which leads to apical prolapse, may also compromise the anterior and posterior compartments. This results in concurrent cystocele, rectocele, and/or enterocele. These often concomitant conditions can lead to urinary incontinence, fecal incontinence, and long-term morbidity [6].

The quantification of prolapse is done clinically using the Pelvic Organ Prolapse Quantification (POP-Q) system, which classifies the prolapse into stages from 0 (no prolapse) to IV [6,8].

Treatment of uterine prolapse is largely dependent on the extent to which a patient is experiencing symptoms. Conservative treatments include pelvic floor muscle training and vaginal pessaries. There are many surgical options for treatment, as well [6,7].

Although it is a common condition, reports exploring the prolonged evolution of symptomatic prolapse, untreated by patient choice, in the context of multiple comorbidities are scarce. This case aims to highlight the role of the family physician in clinical surveillance, shared decision-making, patient advocacy, and appropriate referral [10]*.*

## Case presentation

This case report involves a 70-year-old, retired Caucasian woman who presented with a chief complaint of complete uterine prolapse, associated with significant externalization and episodes of scant abnormal uterine bleeding.

Her relevant medical history includes one pregnancy and one uncomplicated vaginal delivery (G1P1), menopause at 54 years of age (without hormone replacement therapy (HRT)), hypertension, obesity, dyslipidemia, an ischemic stroke (with no identified sequelae), and urinary incontinence.

The uterine prolapse was first identified in primary health care in April 2015, at which time she refused referral for a specialist consultation. In February 2018, she reported worsening symptoms and was referred to the gynecology outpatient clinic. The patient had been referred by her general practitioner in 2018, but she did not have a gynecology consultation until 2019. When she had a gynecology outpatient consultation in 2019, she was assessed and offered corrective surgery. After an explanation of the treatment's risks and benefits, the patient declined the surgery, citing that she did not consider the problem significant in her life. As a result, the patient was discharged from the gynecology clinic, and follow-up was maintained in primary health care. Until July 2025, she was monitored regularly with no worsening of her clinical status, which led her to maintain her refusal of treatment. This was despite various discussions with her family physician addressing the advantages of treatment and countering the "normalization" of prolapse. During this time, the patient always came to her appointments alone, even though she had been asked to bring her daughter or husband with her, in an attempt to involve the family in the decision-making process.

**Figure 1:**
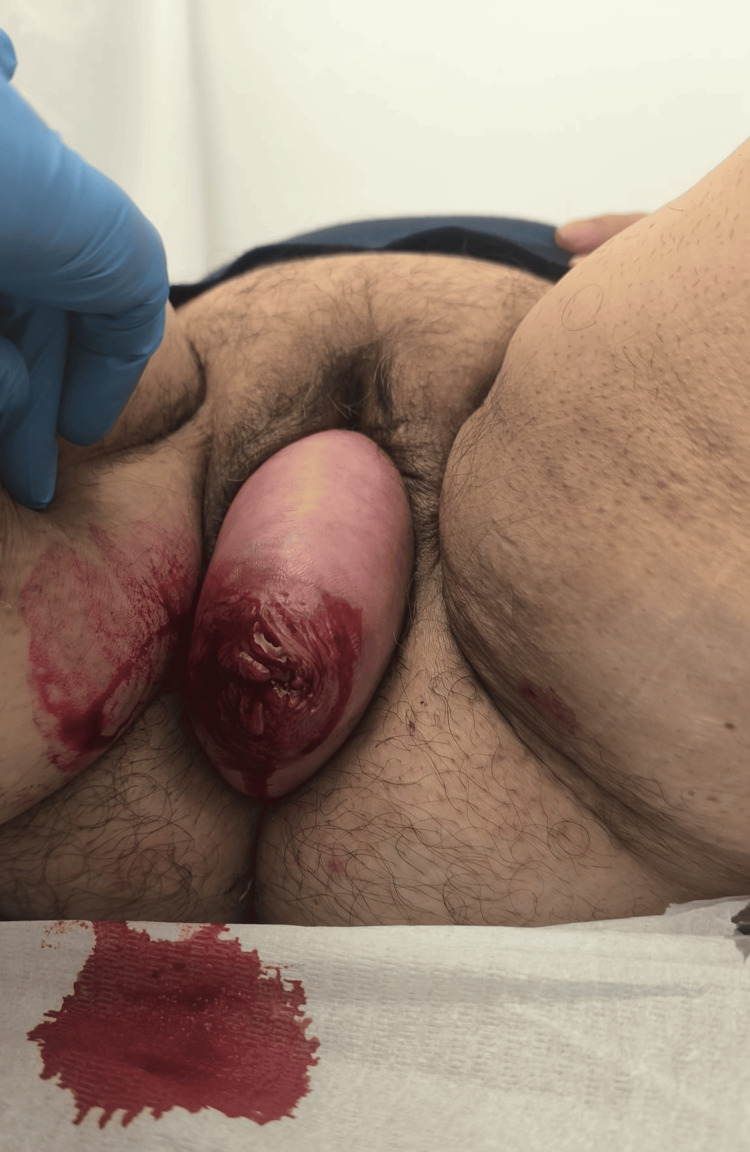
Complete uterine prolapse (stage IV) Total exteriorization of the uterus and cervix through the vaginal introitus is observed. Exposed cervical mucosa with points of active bleeding and areas of erosion.

In July 2025, during a routine consultation, the patient reported, upon questioning, a slight worsening of her usual prolapse and scant abnormal bleeding over the past two months. On physical examination, she was afebrile and hemodynamically stable. The gynecological examination revealed a complete uterine prolapse (stage IV) (Figure 1), which was irreducible in the dorsal decubitus position, presenting with cervical erosion and active bleeding.

A diagnosis of complete uterine prolapse with scant bleeding was established. An attempt at prolapse reduction was unsuccessful, leading to the application of a compressive dressing and referral of the patient to the gynecology emergency department (ED).

In the ED, the patient presented no further active bleeding. Manual reduction of the prolapse was performed; however, it recurred immediately upon the patient standing up. Due to the unavailability of a pessary in the ED, no containment device was placed. She was discharged with instructions for surveillance and local care and was referred to her general practitioner for the subsequent request of a hospital gynecological consultation.

At this stage, the patient consented to the hospital reassessment, demonstrating greater receptivity to the referral, which suggested a positive outlook for treatment adherence. No complications or adverse events were recorded following the acute episode.

In September 2025, at the gynecology outpatient clinic, she was reassessed and surgery was offered again, which the patient declined. The risks and benefits of the available treatments were explained to the patient. Her doubts and questions were clarified, and the patient made an informed decision, opting for pessary placement. An 85 mm pessary was placed, and a follow-up appointment was scheduled for four months later for reassessment and pessary replacement.

## Discussion

The continuity of care in general practice allowed for the early identification of the worsening of a chronic problem that was undervalued by the patient (reported that the symptoms were not significant in her daily life and that they were normal for her age). Recent studies indicate that many women delay seeking treatment for various reasons, including embarrassment, low perception of severity, or considering the symptoms a normal part of aging [11-14]. 

The initial therapeutic refusal, recorded in 2019, must be contextualized within the patient's decision-making process, but it mandates heightened clinical surveillance to ensure safety, given the progressive risk of complications such as bleeding, ulceration, or infection in long-standing prolapses [7]. Shared decision-making (including the family whenever possible, with the patient's consent) is essential in contexts of multiple comorbidities and advanced age, where surgical risk must be weighed [15]. Although surgery is the most effective definitive treatment for advanced prolapse, its economic cost and patient preference warrant discussion [9,16,17]. Even after being properly informed, the patient chose not to undergo surgical correction. This decision must be respected by the physician, who should maintain appropriate and regular follow-up and support. Active listening, regular objective examination, and systematic assessment of the functional impact of the prolapse are fundamental in general practice [10].

Although the signs of worsening were promptly detected and the patient was promptly referred to gynecology, given her age and medical history, a multidisciplinary intervention (including physiotherapy and nutritionist) could have prevented the progression of the problem and/or increased treatment adherence [18-20].

A limitation identified in the course of this clinical case, or, more accurately, a further delay in resolving the patient's complaints, was caused by the lack of technical resources in the ED for non-definitive treatment, such as the absence of pessaries. Pessary use is a viable and effective alternative for many women with advanced prolapse, serving either as definitive treatment or as a bridge to surgery, as demonstrated by recent systematic reviews [15]. The family physician's role in risk reassessment and timely referral was decisive, even when the problem was already known. 

## Conclusions

This case illustrates the challenges of managing advanced uterine prolapse in a context of previous therapeutic refusal and a relevant medical history. Continuous surveillance in general practice was decisive for the timely identification of warning signs (hemorrhage and worsening) and coordination of hospital care. This management underscores the importance of longitudinal follow-up, shared decision-making, and patient advocacy to ensure safety and response to exacerbations. To achieve this, it is essential to inform the patient and family about the risks and benefits of the therapeutic options (non-surgical vs. surgical), with family support being essential in this process.
